# Nutritional vulnerability and its associated characteristics among the elderly in Seoul: analysis of data from the Seoul food survey 2024

**DOI:** 10.3389/fnut.2026.1662335

**Published:** 2026-02-26

**Authors:** Misung Lee, Youngmin Nam, Hye-Jong Yoo, Jihyun Yoon

**Affiliations:** 1 Department of Food and Nutrition, Seoul National University, Seoul, Republic of Korea; 2 Research Institute of Human Ecology, Seoul National University, Seoul, Republic of Korea; 3 Institute on Aging, Seoul National University, Seoul, Republic of Korea

**Keywords:** aged, dietary characteristics, dietary quality, Nutrition Quotient (NQ), Seoul

## Abstract

**Background:**

With South Korea transitioning into a super-aged society in 2024, nutritional vulnerability among the elderly is a growing concern, particularly in Seoul with its large elderly population. This study aimed to identify nutritionally vulnerable elderly individuals in Seoul and examine associated sociodemographic and dietary characteristics using the Nutrition Quotient for the Elderly (NQ-E).

**Methods:**

This study analyzed data on 720 elderly individuals aged 65 years or older from the raw data of the Seoul Food Survey 2024. Based on their scores calculated using NQ-E, respondents were categorized into high, medium, and low grades. In this study, individuals in the low grade were defined as the nutritionally vulnerable group, while those in the medium and high grades were classified as the non-vulnerable group to facilitate analysis of group-specific characteristics. Sociodemographic and dietary characteristics of the two groups were compared. Logistic regression was conducted to identify these characteristics associated with nutritional vulnerability.

**Results:**

A total of 19.0% of respondents were classified as nutritionally vulnerable. Logistic regression analysis revealed, among the total elderly population, men were more likely to be nutritionally vulnerable than women (OR = 2.88, 95% CI: 1.29–6.44). Those with middle school graduation or less had higher odds of nutritional vulnerability compared to those with high school graduation or higher (OR = 3.42, 95% CI: 1.52–7.70). Higher food literacy was associated with lower odds of nutritional vulnerability across all groups: total elderly population (OR = 0.90, 95% CI: 0.87–0.93), elderly men (OR = 0.91, 95% CI: 0.86–0.96), and elderly women (OR = 0.88, 95% CI: 0.83–0.93). Among elderly men, those with lower educational level (middle school graduation or less) were more likely to be nutritionally vulnerable (OR = 8.63, 95% CI: 2.63–28.26), and those living alone were more likely to be nutritionally vulnerable compared to those living with others (OR = 3.42, 95% CI: 1.14–10.27).

**Discussion:**

These findings underscore the importance of implementing targeted interventions to reduce nutritional vulnerability among older adults in Seoul, particularly elderly men living alone. Future research and policy efforts should focus on food literacy as a potential approach to address nutritional vulnerability among the elderly.

## 1 Introduction

As the global population continues to age, the importance of nutritional management and healthy diet among the elderly is increasingly emphasized (1). The elderly population is particularly susceptible to poor dietary quality and nutritional deficiencies due to physiological aging, reduced functional capacity, and changes in social relationships. Maintaining adequate dietary quality is imperative for the prevention of chronic diseases and the preservation of independence and quality of life in later years (2–4). Furthermore, the early identification of nutritional risks and the subsequent implementation of appropriate dietary interventions have been demonstrated to contribute to a reduction in medical costs and the burden on public health systems (5, 6).

In accordance with this global phenomenon, South Korea has experienced a significant demographic transition. In December 2024, the proportion of individuals aged 65 and over surpassed 20%, thereby officially categorizing the country as a super-aged society (7). As of April 2025, the population of elderly individuals residing in Seoul was approximately 1.84 million, constituting 17.6% of the national elderly population. Consequently, Seoul has the second-largest elderly population in Korea, after Gyeonggi Province (8). Given the rapidly growing elderly population, especially in urban areas, greater policy attention is needed to ensure their dietary health and overall well-being.

The development of effective nutritional interventions for the elderly is predicated on a comprehensive understanding of the characteristics that contribute to their nutritional vulnerability. A body of research conducted in South Korea has previously reported gender-based differences in nutritional vulnerability among older adults when evaluated with the Nutrition Screening Initiative (NSI) (9). Additionally, studies have identified variations in nutrient intake and dietary behaviors according to household type when analyzed using the Korea National Health and Nutrition Examination Survey (KNHANES) (10). In addition to sociodemographic characteristics, dietary characteristics have also been identified as key determinants of nutritional vulnerability among the elderly. Previous studies in the United States have shown that poor food environments and food insecurity are associated with lower dietary quality, as indicated by lower HEI scores and reduced fruit intake (11, 12). Similar associations have been observed in Asian contexts. In Korea, higher levels of food literacy have been linked to a reduced risk of insufficient fruit consumption (13), while studies in Japan and China have demonstrated that food literacy and supermarket accessibility are positively associated with dietary quality and dietary diversity, assessed using the HEI and the Dietary Diversity Score (DDS), respectively (14, 15). Although these findings highlight the relevance of food literacy and the food environment to nutritional vulnerability, commonly used assessment tools such as the HEI and NSI were developed based on the dietary patterns of the elderly in the United States. As has been noted in previous research, the dietary practices and cultural context of the elderly in Korea differ from those of the U.S. elderly, limiting the applicability of these tools to the Korean elderly population (16). This gap underscores the need for a culturally appropriate and standardized tool to systematically identify nutritionally vulnerable elderly populations in Korea.

In response to this need, the Ministry of Food and Drug Safety and the Korean Nutrition Society collaboratively developed the Nutrition Quotient for the Elderly (NQ-E), a standardized tool intended to measure the dietary quality and eating behaviors of the elderly population in Korea. Its extensive utilization in both research and practice has been demonstrated in the diagnosis of nutritional status and the development of interventions aimed at improving the eating behaviors of the elderly (17). Given its utility, NQ-E also provides a useful basis for identifying nutritionally vulnerable groups and analyzing characteristics that contribute to nutritional vulnerability.

Despite the practical value of NQ-E, few studies have applied this tool to examine the characteristics of nutritionally vulnerable elderly populations in urban settings such as Seoul. A study of the elderly aged 65–80 years residing in Seoul and Gyeonggi Province reported significant differences in NQ-E scores by gender, age, and use of dietary supplements. Male, older, and non-supplement users exhibited a higher likelihood of being nutritionally vulnerable (18). A similar study of elderly participants attending senior community centers in Seoul found that male older adults scored lower than females in the moderation and dietary behavior domains of the NQ-E suggesting that male older adults are more likely to be nutritionally vulnerable (19). Notably, research conducted in Korea has been limited in examining the associations between nutritional vulnerability and specific dietary characteristics, such as the food environment and food literacy. To address this gap, the objective of this study was to identify the sociodemographic and dietary characteristics associated with nutritional vulnerability among older adults in Seoul, as determined by their NQ-E grades. Identifying the sociodemographic and dietary characteristics associated with nutritional vulnerability among the elderly provides essential evidence for the development of effective nutrition-support policies and constitutes a critical foundation for public health strategies in rapidly aging urban contexts.

## 2 Materials and methods

### 2.1 Data source

The present study analyzed microdata from the Seoul Food Survey 2024, which was designed to represent the Seoul population with a sampling error of 2.2% at the 95% confidence level. The survey is designated as a National Approved Statistic (no. 201011), ensuring verified sampling design and representativeness, and it received ethical approval from the Institutional Review Board of the Korean National Institute for Bioethics Policy (approval no. P01-202409-01-014). The Seoul Food Survey 2024 consisted of 74 questions, including 67 items across five parts— food intake, dietary practice, food purchasing, food safety, and food policies and programs—and 7 items on respondents’ demographic characteristics. For the present analysis, we utilized a subset of items from the food intake, dietary practice, and food purchasing sections (Supplement 1). The survey is conducted on an annual basis among Seoul residents aged 18 and over. The objective of the survey is to analyze citizens’ perceptions and consumption behaviors related to food and to provide the basis for establishing food policies in response to the changing demographic and social structure.

The survey was conducted from September 19 to October 25, 2024. The data were collected through a series of face-to-face interviews administered by trained investigators who visited the participating households. Depending on participants’ circumstances, responses were obtained using either tablet PC–assisted personal interviews (TAPI) or paper-assisted personal interviews (PAPI). Follow-up telephone interviews were conducted to address missingness identified during the initial interview, resulting in a complete dataset. The sample for the Seoul Food Survey 2024 was selected using a stratified cluster sampling design based on the Seoul Survey Database—a repository integrated from resident registration and building records. A total of 2,000 households were initially sampled. Of these, responses were obtained from 76% of the originally selected households, while non-responding households were replaced using a substitution sample. As a result, data from 3,435 individuals were collected, including 733 respondents aged 65 years and older. Consistent with the survey design, multiple individuals from the same household could be included in the analytic sample. To minimize non-response bias, supplementary households were randomly drawn and included as substitute samples. In this study, 13 respondents who selected “no purchase experience” for the food environment satisfaction item were excluded, as no valid satisfaction score could be assigned. This exclusion was made to maintain a consistent sample size across analyses. Consequently, the final analysis encompassed data from 720 elderly respondents.

### 2.2 Measures

#### 2.2.1 Nutritional vulnerability

In this study, the Nutrition Quotient for the Elderly (NQ-E), revised in 2021, was utilized as a nutrition assessment tool to evaluate nutritional vulnerability among the elderly. NQ-E is a validated tool developed to comprehensively assess the dietary quality and eating behaviors of the elderly population in South Korea (20). It was developed based on a large-scale nationwide survey of 1,000 elderly individuals, and its construct validity and reliability have been confirmed.

NQ-E consists of 17 items grouped into three domains: balance, moderation, and practice (Supplement 2). Each item is measured on a 5-point Likert scale, and the Nutrition Quotient (NQ) scores were calculated using the Nutrition Quotient Scoring Program (Excel-based) provided by the Ministry of Food and Drug Safety Korea website (21), which converts responses into standardized 0–100 scores. Specifically, for balance and practice items, the scores are calculated as (response–1) × 100÷4, while for moderation items, the scores are calculated as (5–response) × 100÷4.

The balance domain includes eight items evaluating the intake frequency of essential food groups: fruits, milk or dairy products, fish or shellfish, eggs, beans or bean products, nuts, mixed grains, and water that were selected based on the Dietary Reference Intakes for Koreans (2020), the National Health Promotion Plan (2030), and the Dietary Guidelines for Koreans (2021). This domain reflects the diversity and adequacy of overall nutrient intake. The moderation domain consists of two items assessing the intake frequency of foods prone to overconsumption among the elderly, specifically sweetened snacks or beverages and fatty breads, indicating the extent to which such consumption is restricted. The practice domain includes nine items measuring safe and health-promoting eating behaviors, such as efforts to maintain a healthy diet, expiration date and nutrition labeling check, washing hands before meals, and factors related to physical and mental health, including difficulties in chewing foods, depressed condition, degree of sound sleep, and level of awareness of one’s own health.

During the scoring process, item scores are converted to a 0–100 scale and then aggregated into domain scores using predetermined weights. The three domain scores are then combined to calculate the total NQ-E score, with balance accounting for 55%, moderation for 10%, and practice for 35%. Total and domain scores were categorized into three grades—high (75.0–100%), medium (25.0–74.9%), and low (0–24.9%)—according to the official NQ-E grading criteria provided by the Ministry of Food and Drug Safety. These cutoffs were established based on a national survey of 1,000 elderly individuals residing in Korea, with score ranges determined by percentile distribution (20). In this study, individuals in the low grade were defined as nutritionally vulnerable, while those in the medium and high grades were classified as non-vulnerable.

#### 2.2.2 Sociodemographic and dietary characteristics

The sociodemographic characteristics included sex, age group, educational level, household type, occupation, and monthly household income which were selected according to the objectives of this study. Dietary characteristics included breakfast frequency, high-risk alcohol consumption, food literacy, digital food literacy, and food environment satisfaction.

Based on the number of times breakfast was consumed per week, breakfast frequency was classified into the following categories: “Rarely or never,” “1–2 times/week,” “3–4 times/week,” “5–6 times/week,” and “Daily.” High-risk alcohol consumption, defined as consuming seven or more standard drinks for men or five or more for women on a single occasion, was categorized as: “Non-drinker” (no alcohol consumption in the past year), “<1 time/month,” “1 time/month,” “2–4 times/month,” “≥2 times/week”.

Food literacy refers to an individual’s ability to select, prepare, and manage food to promote a healthy lifestyle, while understanding the cultural, social, and ecological values of and its assessment tool has been validated for reliability and validity (22). Food literacy is composed of three domains: health (14 items assessing knowledge and skills related to nutrition and food safety), enjoyment (8 items assessing attitudes toward food culture and culinary activities), and value (11 items assessing awareness and practices regarding the social and environmental values of food) (Supplement 3). Each question asks respondents to respond on a 5-point Likert scale (1 = strongly not true, 5 = strongly true) to the statement “Please mark the one that is closest to your case.” Scores for each domain were standardized out of 100, and the mean score of the three domains was calculated as the overall food literacy score.

Food digital literacy refers to the ability to order and purchase food using digital technology. It is assessed using three items: online food purchase, use of self-service kiosk in restaurants, use of food delivery application (App) (Supplement 4). Each item is rated on a 5-point Likert scale (1 = strongly disagree, 5 = strongly agree). The mean value of the three items was calculated as the food digital literacy score. The scores were analyzed on the original 5-point scale without transformation.

Food environment satisfaction is an indicator of satisfaction with the local food environment and consists of five items: availability, accessibility, affordability, convenience, and acceptability (Supplement 5). This construct has been shown in previous studies to be measured with validated tools demonstrating acceptable reliability and validity (23). Each item is measured on a 5-point Likert scale (1 = strongly disagree, 5 = strongly agree). The mean value of the five items is used as the food environment satisfaction score. The scores were analyzed on the original 5-point scale without transformation.

### 2.3 Data analysis

A complex sample analysis was applied by incorporating sampling weights, stratification variables, and the primary sampling unit (PSU), defined at the enumeration-area level. The Rao–Scott chi-square test was used for categorical variables (sex, age group, educational level, household type, occupation, monthly household income, breakfast frequency, and high-risk drinking frequency), and the general linear model (GLM) was applied for continuous variables (food literacy, food digital literacy, and food environment satisfaction). As described earlier, the NQ-E were categorized into three levels: high (75.0–100%), medium (25.0–74.9%), and low (0–24.9%). The distribution of NQ-E grades was examined, and domain-specific NQ-E scores were compared across these grade levels. To identify characteristics associated with nutritional vulnerability, unadjusted and adjusted logistic regression analyses were conducted separately for the total elderly population, elderly men, and elderly women in Seoul. Unadjusted analyses were first performed using univariate logistic regression models to examine the association between each sociodemographic and dietary characteristic and nutritional vulnerability. Variables that showed statistical significance in the unadjusted analyses were selected for inclusion in the adjusted multivariable logistic regression models.

During the process of constructing the adjusted models, monthly household income was initially examined in the unadjusted analyses. Measured at the household level as average monthly income, household income was closely related to household type. When both variables were included in the adjusted models, the association of household type was attenuated without a meaningful improvement in model fit. Accordingly, household type was retained in the final adjusted models as a more direct indicator of living conditions relevant to nutritional vulnerability.

The dependent variable was nutritional vulnerability status, coded as 0 for nutritionally non-vulnerable and 1 for nutritionally vulnerable. The independent variables included gender, age group, education level, household type, occupation, monthly household income food literacy, digital food literacy, and food environment satisfaction. Food literacy, digital food literacy, and food environment satisfaction were treated as continuous variables, whereas all other variables were treated as categorical.

Categorical variables were presented as unweighted frequencies and weighted percentages, and continuous variables were reported as weighted means and standard errors (SE). Logistic regression results were expressed as odds ratios (ORs) with 95% confidence intervals (CIs). All analyses were conducted using IBM SPSS Statistics version 29.0 (Armonk, NY, United States), and statistical significance was set at *α* = 0.05.

## 3 Results

### 3.1 Sociodemographic characteristics of the respondents

Table 1 presents the sociodemographic characteristics of the respondents; *p*-values indicate gender differences in proportional distributions based on the Rao–Scott χ^2^ test. The proportions of men and women were 44.5 and 55.5%, respectively. About 3/4 of the respondents were aged between 65 and 74 years. A total of 58.6% lived with others, while 41.4% lived alone. More than half of the respondents (57.5%) had a high school graduation or higher. Approximately 40% of the respondents were employed, and 34.3% had a monthly household income below 2,000,000 Korean Won (KRW). Household income was analyzed in three categories (<2 million KRW, 2–3.5 million KRW, ≥3.5 million KRW), reflecting the ranges provided in the Seoul Food Survey.

**Table 1:** Sociodemographic characteristics of the respondents.

Characteristics	Total (*n* = 720)	Men (*n* = 336)	Women (*n* = 384)	*p*-value^a^
Age group (yrs)				
65–74	606 (75.6)	287 (82.1)	319 (70.4)	0.087
≥ 75	114 (24.4)	49 (17.9)	65 (29.6)	
Education level				
Middle school graduation or less	133 (42.5)	39 (31.2)	94 (51.6)	0.015
High school graduation or higher	587 (57.5)	297 (68.8)	290 (48.4)	
Household type				
Living alone	206 (41.4)	55 (24.3)	151 (55.0)	<0.001
Living with others	514 (58.6)	281 (75.7)	233 (45.0)	
Occupation				
Employed	333 (42.4)	239 (70.8)	94 (19.7)	<0.001
Others (housewife, unemployed, etc.)	387 (57.6)	97 (29.2)	290 (80.3)	
Monthly household income (10,000 KRW)				
< 200	159 (34.3)	44 (18.6)	115 (47.0)	<0.001
200 to < 350	296 (35.4)	146 (39.6)	150 (32.0)	
≥ 350	265 (30.3)	146 (41.8)	119 (21.1)	

Values are presented as unweighted numbers (weighted %). All weighted models accounted for the complex sampling design of the Seoul Food Survey 2024.

a By Rao-Scott χ2 test.

Multiple characteristics significantly differed by gender. The proportion of elderly men with high school graduation or higher was 68.8%, which was higher than that of elderly women (48.4%). The proportion of employed individuals was 70.8% among men and 19.7% among women. Regarding monthly household income, 18.6% of elderly men had an income of less than 2 million KRW, whereas the proportion was higher among elderly women (47.0%). The proportion of those living alone was significantly higher among elderly women (55.0%) compared to elderly men (24.3%).

Table 2 presents the distribution of the NQ-E grades; *p*-values reflect gender differences in the proportion of participants across NQ-E categories, tested using the Rao–Scott χ^2^ test. A significant gender difference was observed in the distribution of the NQ-E grades (*p* = 0.041), with a higher proportion of elderly men classified as low grade (26.3%) compared to elderly women (13.1%), representing approximately a twofold difference. In the balance domain, a significant gender difference was also identified (*p* < 0.001), with 26.4% of elderly men classified as low grade, compared to 8.1% of elderly women. No significant gender differences were observed in the distribution of grades in the moderation and practice domains. Detailed domain-specific NQ-E scores are provided in Appendix A.

**Table 2:** Distribution of NQ-E grades among the elderly in Seoul.

Items	Total (*n* = 720)	Men (*n* = 336)	Women (*n* = 384)	*p*-value^a^
NQ-E				
High	316 (37.1)	134 (34.0)	182 (39.7)	0.041
Medium	321 (43.9)	154 (39.7)	167 (47.2)	
Low	83 (19.0)	48 (26.3)	35 (13.1)	
Balance				
High	281 (34.6)	110 (26.0)	171 (41.5)	<0.001
Medium	356 (49.1)	174 (47.6)	182 (50.3)	
Low	83 (16.3)	52 (26.4)	31 (8.1)	
Moderation				
High	217 (29.6)	94 (28.1)	123 (30.8)	0.840
Medium	362 (53.2)	180 (53.7)	182 (52.8)	
Low	141 (17.2)	62 (18.2)	79 (16.3)	
Practice				
High	269 (27.7)	121 (28.9)	148 (26.7)	0.819
Medium	322 (43.5)	157 (41.3)	165 (45.3)	
Low	129 (28.8)	58 (29.8)	71 (28.0)	

NQ-E, nutrition quotient for the elderly. Values are presented as unweighted numbers (weighted %). All weighted models accounted for the complex sampling design of the Seoul Food Survey 2024. NQ-E and its domain scores were classified into three levels—low (0–24.9%), medium (25.0–74.9%), and high (75.0–100%)—based on the national distribution of NQ-E scores among the elderly.

a Rao-Scott χ2 test.

### 3.2 Sociodemographic and dietary characteristics according to nutritional vulnerability among the elderly in Seoul

Table 3 presents the sociodemographic characteristics of the elderly in Seoul by nutritional vulnerability. Among the total elderly population, significant differences were found in gender (*p* = 0.023) and education level (*p* < 0.001). The proportion of men was higher in the nutritionally vulnerable group (61.7%) compared to the non-vulnerable group (40.4%). Similarly, the nutritionally vulnerable group showed a higher proportion of individuals with middle school graduation or less (66.3%) compared to the non-vulnerable group (36.9%).

**Table 3:** Sociodemographic characteristics of the elderly in Seoul by nutritional vulnerability.

Characteristics	Total				Men				Women			
	Total (*n* = 720)	Nutritionally vulnerable group^a^ (*n* = 83)	Nutritionally non-vulnerable group^b^ (*n* = 637)	*p*-value^c^	Total (*n* = 336)	Nutritionally vulnerable group^a^ (*n* = 48)	Nutritionally non-vulnerable group^b^ (*n* = 288)	*p*- value^c^	Total (*n* = 384)	Nutritionally vulnerable group^a^ (*n* = 35)	Nutritionally non-vulnerable group^b^ (*n* = 349)	*p*-value^c^
Gender												
Men	336 (44.5)	48 (61.7)	288 (40.4)	0.023	-				-			
Women	384 (55.5)	35 (38.3)	349 (59.6)									
Age group (yrs)												
65–74	606 (75.6)	63 (71.7)	543 (76.6)	0.524	287 (82.1)	37 (69.3)	250 (86.7)	0.034	319 (70.4)	26 (75.4)	293 (69.7)	0.488
≥ 75	114 (24.4)	20 (28.3)	94 (23.4)		49 (17.9)	11 (30.7)	38 (13.3)		65 (29.6)	9 (24.6)	56 (30.3)	
Education level												
Middle school graduation or less	133 (42.5)	34 (66.3)	99 (36.9)	<0.001	39 (31.2)	17 (66.6)	22 (18.5)	<0.001	94 (51.6)	17 (65.8)	77 (49.4)	0.201
High school graduation or higher	587 (57.5)	49 (33.7)	538 (63.1)		297 (68.8)	31 (33.4)	266 (81.5)		290 (48.4)	18 (34.2)	272 (50.6)	
Household type												
Living alone	206 (41.4)	37 (50.1)	169 (39.3)	0.191	55 (24.3)	17 (43.3)	38 (17.5)	0.003	151 (55.0)	20 (61.1)	131 (54.1)	0.541
Living with others	514 (58.6)	46 (49.9)	468 (60.7)		281 (75.7)	31 (56.7)	250 (82.5)		233 (45.0)	15 (38.9)	218 (45.9)	
Occupation												
Employed	333 (42.4)	31 (39.9)	302 (43.0)	0.726	239 (70.8)	28 (57.9)	211 (75.4)	0.042	94 (19.7)	3 (10.8)	91 (21.0)	0.283
Others (housewife, unemployed, etc.)	387 (57.6)	52 (60.1)	335 (57.0)		97 (29.2)	20 (42.1)	77 (24.6)		290 (80.3)	32 (89.2)	258 (79.0)	
Monthly household income (10,000 KRW)												
< 200	159 (34.3)	38 (49.2)	121 (30.8)	0.113	44 (18.6)	16 (40.9)	28 (10.6)	0.004	115 (47.0)	22 (62.7)	93 (44.6)	0.116
200 to < 350	296 (35.4)	23 (29.3)	273 (36.8)		146 (39.6)	15 (27.8)	131 (43.8)		150 (32.0)	8 (31.6)	142 (32.0)	
≥ 350	265 (30.3)	22 (21.5)	243 (32.4)		146 (41.8)	17 (31.3)	129 (45.6)		119 (21.1)	5 (5.7)	114 (23.4)	

Values are presented as unweighted numbers (weighted %). All weighted models accounted for the complex sampling design of the Seoul Food Survey 2024.

a Group classified as “low” (0–24.9%) based on the Nutrition Quotient (NQ-E) score, representing the nutritionally vulnerable group.

b Group classified as “medium” (25.0–74.9%) or “high” (75.0–100%) based on the NQ-E score, representing the non-vulnerable group.

c Rao-Scott χ^2^ test.

Among elderly men, significant differences were observed in age group (*p* = 0.034), education level (*p* < 0.001), household type (*p* = 0.003), occupation (*p* = 0.042), and monthly household income (*p* = 0.004). Compared to the non-vulnerable group, nutritionally vulnerable men were more likely to be aged ≥75 years, have middle school education or less, live alone, be unemployed or in other categories, and have a monthly household income <2 million KRW. Conversely, non-vulnerable men were more likely to be aged 65–74 years, have high school education or higher, live with others, be employed, and have a monthly household income ≥3.5 million KRW.

Table 4 presents the dietary characteristics of the elderly in Seoul by nutritional vulnerability. In all groups, including the total elderly population, elderly men, and elderly women, no statistically significant differences were observed in breakfast frequency or high-risk alcohol consumption between the nutritionally vulnerable and non-vulnerable groups.

**Table 4:** Dietary characteristics of the elderly in Seoul by nutritional vulnerability.

Characteristics	Total				Men				Women			
	Total (*n* = 720)	Nutritionally vulnerable group^a^ (*n* = 83)	Nutritionally non-vulnerable group^b^ (*n* = 637)	*p*-value^c^	Total (*n* = 336)	Nutritionally vulnerable group^a^ (*n* = 48)	Nutritionally non-vulnerable group^b^ (*n* = 288)	*p*-value^c^	Total (*n* = 384)	Nutritionally vulnerable group^a^ (*n* = 35)	Nutritionally non-vulnerable group^b^ (*n* = 349)	*p*-value^c^
Breakfast frequency												
Rarely or never	31 (4.6)	11 (8.9)	20 (3.6)	0.064	17 (4.6)	7 (6.0)	10 (4.1)	0.084	14 (4.6)	4 (13.5)	10 (3.3)	0.158
1 ~ 2 times/week	70 (9.5)	12 (14.1)	58 (8.5)		35 (13.5)	8 (19.9)	27 (11.2)		35 (6.3)	4 (4.7)	31 (6.6)	
3 ~ 4 times/week	140 (14.3)	9 (6.9)	131 (16.1)		63 (13.9)	6 (4.3)	57 (17.3)		77 (14.7)	3 (11.1)	74 (15.2)	
5 ~ 6 times/week	127 (14.0)	12 (8.3)	115 (15.3)		69 (16.1)	6 (8.3)	63 (18.8)		58 (12.3)	6 (8.2)	52 (12.9)	
Daily	352 (57.6)	39 (61.9)	313 (56.6)		152 (52.0)	21 (61.5)	131 (48.5)		200 (62.1)	18 (62.6)	182 (62.1)	
High-risk alcohol consumption^d^												
Non drinker	297 (51.3)	39 (56.3)	258 (50.1)	0.898	80 (31.5)	14 (42.5)	66 (27.5)	0.583	217 (67.1)	25 (78.6)	192 (65.4)	0.486
<1 time/month	215 (22.1)	21 (19.2)	194 (22.8)		114 (28.3)	16 (24.2)	98 (29.7)		101 (17.2)	5 (11.3)	96 (18.1)	
2 ~ 4 times/month	146 (17.0)	16 (15.8)	130 (17.3)		97 (24.7)	12 (20.2)	85 (26.2)		49 (10.9)	4 (8.6)	45 (11.2)	
≥2 times/week	62 (9.6)	7 (8.7)	55 (9.8)		45 (15.6)	6 (13.2)	39 (16.5)		17 (4.8)	1 (1.5)	16 (5.3)	
Food literacy^e^	56.18 ± 1.0	50.34 ± 2.0	62.03 ± 0.6	<0.001	56.24 ± 1.3	51.09 ± 2.5	61.40 ± 0.9	<0.001	55.85 ± 1.6	48.16 ± 3.3	63.55 ± 0.8	<0.001
Health	55.88 ± 1.3	49.26 ± 2.4	62.49 ± 0.8	<0.001	56.18 ± 1.5	51.07 ± 3.0	61.29 ± 1.2	<0.001	54.64 ± 1.7	44.78 ± 3.4	64.49 ± 0.9	<0.001
Enjoyment	51.35 ± 1.1	45.70 ± 2.1	57.00 ± 0.6	<0.001	51.07 ± 1.2	45.75 ± 2.4	56.40 ± 0.9	<0.001	51.95 ± 1.9	44.94 ± 3.8	58.97 ± 0.9	<0.001
Value	61.33 ± 1.2	56.05 ± 2.2	66.61 ± 0.8	<0.001	61.47 ± 1.6	56.44 ± 3.0	66.50 ± 1.1	0.004	60.97 ± 1.8	54.77 ± 3.5	67.18 ± 1.0	0.001
Digital food literacy^f^	2.16 ± 0.1	1.98 ± 0.1	2.34 ± 0.1	<0.001	2.52 ± 0.1	2.33 ± 0.2	2.71 ± 0.1	<0.001	1.89 ± 0.1	1.60 ± 0.2	2.19 ± 0.1	0.012
Online food purchase	2.07 ± 0.1	1.92 ± 0.1	2.22 ± 0.1	<0.001	2.40 ± 0.1	2.26 ± 0.2	2.54 ± 0.1	<0.001	1.83 ± 0.1	1.56 ± 0.2	2.11 ± 0.1	0.029
Use of self-service kiosk in restaurants	2.38 ± 0.1	2.14 ± 0.1	2.62 ± 0.1	<0.001	2.78 ± 0.1	2.50 ± 0.2	3.06 ± 0.1	<0.001	2.06 ± 0.2	1.71 ± 0.3	2.40 ± 0.1	0.014
Use of food delivery application (App)	2.04 ± 0.1	1.89 ± 0.1	2.19 ± 0.1	<0.001	2.37 ± 0.1	2.23 ± 0.2	2.51 ± 0.1	<0.001	1.79 ± 0.1	1.52 ± 0.2	2.07 ± 0.1	0.008
Food environment satisfaction^g^	3.70 ± 0.0	3.52 ± 0.1	3.89 ± 0.0	<0.001	3.75 ± 0.1	3.63 ± 0.1	3.87 ± 0.1	0.012	3.64 ± 0.1	3.36 ± 0.2	3.92 ± 0.0	<0.001
Availability	3.89 ± 0.1	3.82 ± 0.1	3.97 ± 0.1	0.481	3.95 ± 0.1	3.91 ± 0.2	3.98 ± 0.1	0.228	3.81 ± 0.1	3.64 ± 0.2	3.97 ± 0.1	0.097
Accessibility	3.69 ± 0.1	3.46 ± 0.1	3.91 ± 0.1	<0.001	3.69 ± 0.1	3.52 ± 0.1	3.85 ± 0.1	0.028	3.69 ± 0.1	3.43 ± 0.1	3.95 ± 0.1	0.003
Affordability	3.53 ± 0.1	3.26 ± 0.1	3.80 ± 0.0	<0.001	3.61 ± 0.1	3.47 ± 0.2	3.76 ± 0.1	<0.001	3.40 ± 0.1	2.95 ± 0.2	3.84 ± 0.1	<0.001
Convenience	3.65 ± 0.1	3.47 ± 0.1	3.83 ± 0.0	0.073	3.70 ± 0.1	3.56 ± 0.1	3.83 ± 0.1	0.057	3.57 ± 0.1	3.30 ± 0.3	3.84 ± 0.1	0.039
Acceptability	3.76 ± 0.1	3.57 ± 0.1	3.95 ± 0.1	0.011	3.80 ± 0.1	3.68 ± 0.2	3.92 ± 0.1	0.095	3.73 ± 0.1	3.49 ± 0.2	3.97 ± 0.1	0.013

Values are presented as unweighted numbers (weighted %) or mean ±SD. All weighted models accounted for the complex sampling design of the Seoul Food Survey 2024.

a Group classified as “low” (0–24.9%) based on the Nutrition Quotient (NQ-E) score, representing the nutritionally vulnerable group.

b Group classified as “medium” (25.0–74.9%) or “high” (75.0–100%) based on the NQ-E score, representing the non-vulnerable group.

c Rao–Scott χ^2^ test or general linear model (GLM), with adjustment for gender and education level in the total elderly population, and for age group, education level, household type, occupation, and monthly household income in elderly men; unadjusted models were applied for elderly women.

d High-risk alcohol consumption was classified based on the frequency of high-risk drinking, defined as consuming ≥7 standard drinks for men or ≥5 for women on a single occasion. Respondents who reported no such drinking in the past year were categorized as “non drinker”.

e Calculated as the mean score of the three food literacy items: health (14 items), enjoyment (8 items), and value (11 items) (out of 100 points).

f Calculated as the mean score of the three digital food literacy items: online food purchasing, use of self-service kiosk, and use of food delivery application (out of five points using a five-point Likert scale).

g Calculated as the mean score of the five food environment satisfaction items: availability, accessibility, affordability, convenience, and acceptability (out of five points using a five-point Likert scale).

In the total elderly population, group differences were examined using general linear model (GLM) with adjustment for gender and education level, which were selected as covariates based on sociodemographic variables that differed significantly between the nutritionally vulnerable and non-vulnerable groups in Table 3. After adjustment, the nutritionally vulnerable group showed significantly lower food literacy scores compared to the non-vulnerable group (nutritionally vulnerable: 50.3 points, non-vulnerable: 62.0 points, *p* < 0.001) across all domains, including health, enjoyment, and value. The largest difference was observed in the health domain (nutritionally vulnerable group: 49.3 points, non-vulnerable group: 62.5 points, *p* < 0.001). Digital food literacy was significantly lower in the nutritionally vulnerable group than in the non-vulnerable group (nutritionally vulnerable: 2.0 points, non-vulnerable: 2.3 points, *p* < 0.001), with significantly lower scores observed for all three items: online food purchase, use of self-service kiosks in restaurants, and use of food delivery applications. The greatest difference was observed in the use of self-service kiosks in restaurants (nutritionally vulnerable group: 2.1 points, non-vulnerable group: 2.6 points, *p* < 0.001). Food environment satisfaction was significantly lower in the nutritionally vulnerable group (nutritionally vulnerable: 3.5 points, non-vulnerable: 3.9 points, *p* < 0.001). In particular, the nutritionally vulnerable group showed lower levels of accessibility (*p* < 0.001), affordability (*p* < 0.001), and acceptability (*p* = 0.011) compared to the non-vulnerable group.

Among elderly men, general linear model (GLM) were applied with adjustment for age group, education level, household type, occupation, and monthly household income. The nutritionally vulnerable group showed significantly lower food literacy scores compared to the non-vulnerable group (nutritionally vulnerable group: 51.1 points, non-vulnerable group: 61.4 points, *p* < 0.001) across all domains. The largest difference was observed in the enjoyment domain (nutritionally vulnerable group: 45.7 points, non-vulnerable group: 56.4 points, *p* < 0.001). Digital food literacy was also significantly lower in the nutritionally vulnerable men compared to the non-vulnerable men (nutritionally vulnerable group: 2.3 points, non-vulnerable group: 2.7 points, *p* < 0.001), with all three items showing significantly lower scores. The greatest difference was observed in the use of self-service kiosks in restaurants (nutritionally vulnerable group: 2.5 points, non-vulnerable group: 3.1 points, *p* < 0.001). Food environment satisfaction was significantly lower in the nutritionally vulnerable group (nutritionally vulnerable group: 3.6 points, non-vulnerable group: 3.9 points, *p* = 0.012). In particular, the nutritionally vulnerable group showed lower levels of accessibility (*p* = 0.028) and affordability (*p* < 0.001) compared to the non-vulnerable group.

Among elderly women, group differences were examined using a general linear model (GLM) without covariate adjustment. The nutritionally vulnerable group showed significantly lower food literacy scores compared to the non-vulnerable group (nutritionally vulnerable group: 48.2 points, non-vulnerable group: 63.6 points, *p* < 0.001) across all domains. The largest difference was observed in the health domain (nutritionally vulnerable group: 44.8 points, non-vulnerable group: 64.5 points, *p* < 0.001). Digital food literacy was also significantly lower in the nutritionally vulnerable group (nutritionally vulnerable group: 1.6 points, non-vulnerable group: 2.2 points, *p* = 0.012), with significantly lower scores observed for all three items. The greatest difference was observed in the use of self-service kiosks in restaurants (nutritionally vulnerable group: 1.7 points, non-vulnerable group: 2.4 points, *p* = 0.014). Food environment satisfaction was significantly lower in the nutritionally vulnerable group (nutritionally vulnerable group: 3.4 points, non-vulnerable group: 3.9 points, *p* < 0.001). In particular, the nutritionally vulnerable group showed lower levels of accessibility (*p* = 0.003), affordability (*p* < 0.001), convenience (*p* = 0.039), and acceptability (*p* = 0.013) compared to the non-vulnerable group.

### 3.3 Characteristics associated with nutritional vulnerability among the elderly in Seoul

Table 5 presents the results of unadjusted and adjusted logistic regression analyses examining the sociodemographic and dietary characteristics associated with nutritional vulnerability among the elderly in Seoul. In the total elderly population, gender, educational level, and food literacy were significantly associated with nutritional vulnerability. Specifically, men were significantly more likely to be nutritionally vulnerable than women (OR = 2.88, 95% CI: 1.29–6.44, *p* = 0.010). Elderly individuals with middle school graduation or less were more likely to be nutritionally vulnerable compared to those with high school graduation or higher (OR = 3.42, 95% CI: 1.52–7.70, *p* = 0.003). Higher food literacy was inversely associated with nutritional vulnerability (OR = 0.90, 95% CI: 0.87–0.93, *p* < 0.001).

**Table 5:** Sociodemographic and dietary characteristics associated with nutritional vulnerability among the elderly in Seoul.

Characteristics	Total			Men			Women		
	*n*	Unadjusted OR (95% CI)^a^	Adjusted OR (95% CI)^b^	*n*	Unadjusted OR (95% CI)^a^	Adjusted OR (95% CI)^b^	*n*	Unadjusted OR (95% CI)^a^	Adjusted OR (95% CI)^b^
Gender									
Man	336	2.37 (1.12–5.05)^*^	2.88(1.29–6.44)^*^	-			-		
Woman (ref.)	384	1.00	1.00						
Age group (yrs)									
65–74 (ref.)	606	1.00	-	287	1.00	1.00	319	1.00	-
≥ 75	114	1.29 (0.58–2.86)		49	2.88 (1.06–7.87)^*^	0.79 (0.14–4.49)	65	0.75 (0.33–1.70)	
Education level									
Middle school graduation or less	133	3.37 (1.63–6.95)^*^	3.42 (1.52–7.70)^*^	39	8.80 (3.25–23.85)^**^	8.63 (2.63–28.26)^**^	94	1.97 (0.69–5.66)	-
High school graduation or higher (ref.)	587	1.00	1.00	297	1.00	1.00	290	1.00	
Household type									
Living alone	206	1.55 (0.80–2.99)	-	55	3.59 (1.49–8.64)^*^	3.42 (1.14–10.27)^*^	151	1.33 (0.53–3.33)	-
Living with others (ref.)	514	1.00		281	1.00	1.00	233	1.00	
Occupation									
Employed	333	0.88 (0.43–1.80)	-	239	0.45 (0.21–0.98)^*^	0.96 (0.23–4.02)	94	0.46 (0.11–1.98)	-
Others (Housewife, unemployed, etc.) (ref.)	387	1.00		97	1.00	1.00	290	1.00	
Monthly household income (10,000 KRW)									
< 200	159	2.40 (0.87–6.59)	-	44	5.61 (1.69–18.70)^*^	-	115	5.74 (1.21–27.25)^*^	2.66 (0.64–11.08)
200 to < 350	296	1.19 (0.42–3.41)		146	0.92 (0.27–3.21)		150	4.03 (0.72–22.43)	3.36 (0.68–16.58)
≥ 350	265	1.00		146	1.00		119	1.00	1.00
Food literacy^c^	720	0.88 (0.85–0.92)^**^	0.90 (0.87–0.93)^**^	336	0.90 (0.87–0.95)^**^	0.91 (0.86–0.96)^**^	384	0.86 (0.81–0.91)^**^	0.88 (0.83–0.93)^**^
Digital food literacy^d^	720	0.57 (0.37–0.87)^*^	1.01 (0.59–1.73)	336	0.46 (0.26–0.82)^*^	1.17 (0.55–2.52)	384	0.51 (0.26–1.03)	-
Food environment satisfaction^e^	720	0.26 (0.14–0.48)^**^	0.53 (0.24–1.17)	336	0.35 (0.15–0.84)^*^	0.84 (0.26–2.73)	384	0.16 (0.06–0.41)^**^	0.38 (0.16–0.93)^*^

*p < 0.05, **p < 0.01; OR, odds ratio; CI, confidence interval. Model statistics were derived from the same multivariable models: Total elderly, Nagelkerke R^2^ = 0.393, Wald F = 12.151 (*p* < 0.001); elderly men, Nagelkerke R^2^ = 0.462, Wald F = 9.209 (*p* < 0.001); elderly women, Nagelkerke R^2^ = 0.409, Wald F = 12.120 (*p* < 0.001).

a Unadjusted odds ratios were estimated using univariate logistic regression models.

b Adjusted odds ratios were estimated using multivariable logistic regression models including gender, age group, education level, household type, occupation, monthly household income, food literacy, digital food literacy, and food environment satisfaction as covariates.

c Calculated as the mean score of the three food literacy items: health, enjoyment, and value (out of 100 points).

d Calculated as the mean score of the three digital food literacy items: online food purchasing, use of self-service kiosk, and use of food delivery application (out of five points using a five-point Likert scale).

e Calculated as the mean score of the five food environment satisfaction items: availability, accessibility, affordability, convenience, and acceptability (out of five points using a five-point Likert scale).

Among elderly men, educational level, household type, and food literacy were significantly associated with nutritional vulnerability. Those with middle school graduation or less were significantly more likely to be nutritionally vulnerable compared to those with high school graduation or higher (OR = 8.63, 95% CI: 2.63–28.26, *p* < 0.001). Elderly men living alone were more likely to be nutritionally vulnerable compared to those living with others (OR = 3.42, 95% CI: 1.14–10.27, *p* = 0.028). Higher food literacy was associated with a significantly lower risk of nutritional vulnerability (OR = 0.91, 95% CI: 0.86–0.96, *p* < 0.001).

Among elderly women, food literacy and food environment satisfaction were significantly associated with nutritional vulnerability. Higher food literacy was inversely associated with nutritional vulnerability (OR = 0.88, 95% CI: 0.83–0.93, *p* < 0.001), and higher food environment satisfaction also significantly reduced the likelihood of nutritional vulnerability (OR = 0.38, 95% CI: 0.16–0.93, *p* = 0.034).

## 4 Discussion

This study classified elderly individuals in Seoul into nutritionally vulnerable and non-vulnerable groups based on the Nutrition Quotient for the Elderly (NQ-E) and analyzed their associated characteristics. According to the results, 19.0% of Seoul’s elderly population were classified as nutritionally vulnerable, a slightly lower proportion compared to the national distribution (low: 25%, medium: 50%, high: 25%) (19). Despite the lower proportion compared to the national distribution, the fact that approximately 340,000 individuals among Seoul’s elderly population of 1.78 million as of July 2024 are nutritionally vulnerable underscores the considerable scale of the issue and the need for appropriate interventions (8).

Nutritional vulnerability in the elderly is closely linked to food insecurity, a well-established risk factor for poor nutritional status in later life (24, 25). A previous study using the 2023 Seoul Food Survey examined food insecurity among the elderly in Seoul and reported that 56.9% were classified as food insecure, with household type, digital food literacy, and food environment satisfaction identified as key associated characteristics (26). That study assessed food insecurity based on the 2023 survey data, focusing primarily on insufficient food quantity and limited dietary diversity.

Building on this line of research, the present study used data from the 2024 Seoul Food Survey to assess nutritional vulnerability using the Nutrition Quotient for the Elderly (NQ-E), an age-specific composite index encompassing the domains of balance, moderation, and dietary practice. By focusing on dietary behaviors and practices beyond food access and quantity, this study provides complementary evidence that advances understanding of nutritional vulnerability among the elderly in Seoul.

Logistic regression analysis identified gender, education level, and food literacy as significant characteristics associated with nutritional vulnerability in the total elderly population in Seoul. In particular, elderly men were nearly three times more likely to be nutritionally vulnerable compared to elderly women. These results are consistent with previous research analyzing the 7th Korean National Health and Nutrition Examination Survey, which reported that elderly men tend to have poorer dietary quality than women (27).

Food literacy consistently emerged as a significant characteristic associated with nutritional vulnerability across all groups—the total elderly population, elderly men, and elderly women. Previous studies have shown that elderly individuals at risk of nutritional vulnerability often experience difficulties with meal preparation (28, 29). Specific components of food literacy, such as meal preparation skills, healthy snacking habits, and budget management, have been reported to strongly influence dietary quality (30). Moreover, high food literacy has been associated with an approximately 86% reduced risk of insufficient fresh fruit consumption among the elderly (13). These findings highlight the relevance of food literacy in understanding nutritional vulnerability. Nevertheless, due to the cross-sectional design of this study, it cannot be determined whether enhancing food literacy would directly reduce vulnerability. Future research should therefore examine whether interventions aimed at enhancing food literacy can effectively improve nutritional outcomes among the elderly.

Furthermore, the lower food literacy observed among elderly men have been consistently reported in previous studies. A study conducted among the elderly in Seoul and Gyeonggi Province found that 74.1% of elderly men relied on their spouses for meal preparation, with significantly lower rates of independent cooking and food purchasing compared to women (31). Similarly, a study of community-dwelling elderly individuals in Japan reported that elderly men with social vulnerability tended to have lower dietary quality, whereas elderly women maintained their dietary quality regardless of social vulnerability (32). These findings emphasize the need for tailored nutrition support and education programs targeting elderly men, who face greater challenges in maintaining adequate dietary practices due to limited cooking skills and heightened social isolation.

Education level was significantly associated with nutritional vulnerability in the total elderly population and among elderly men, with those having a middle school education or less showing higher odds of vulnerability. This aligns with previous studies indicating that lower education levels are linked to poorer dietary quality and reduced access to nutritious foods due to economic constraints (33–35).

Among elderly men, household type was significantly associated with nutritional vulnerability. Specifically, those living alone had over three times the odds of being nutritionally vulnerable compared to those living with others. Previous studies have reported that elderly men living alone tend to have poorer dietary quality (10, 36, 37). A study using the 2017 Korean Elderly Survey reported that 78.9% of elderly men living alone were at risk of nutritional deficiency, with excessive alcohol use, multiple chronic diseases, and negative health perceptions as contributing factors (36). These findings indicate that elderly men living alone face substantial barriers to maintaining a healthy diet.

In this study, food environment satisfaction—comprising availability, accessibility, affordability, convenience, and acceptability—was significantly associated with nutritional vulnerability only among elderly women. Specifically, nutritionally vulnerable women showed lower satisfaction with accessibility and affordability than those in the non-vulnerable group. These findings are consistent with a prior study of 372 elderly adults in two districts of Seoul, which reported that a perceived lack of nearby food stores doubled the risk of nutritional deficiency, and that perceived unaffordability was also associated with increased nutritional risk (38). A qualitative study conducted at a senior welfare center further showed that low-income elderly individuals often traveled long distances to obtain affordable food, illustrating how physical and economic constraints can jointly limit food accessibility (39). Therefore, to reduce nutritional vulnerability among elderly women, community-level policy interventions aimed at improving food accessibility and affordability are essential—particularly those that address both physical and economic barriers to accessing nutritious food (40).

Based on these findings, gender, educational level, and household type were identified as sociodemographic characteristics significantly associated with nutritional vulnerability among the elderly in Seoul, with the association for household type observed specifically among elderly men. Although both gender and education level demonstrated significant associations with nutritional vulnerability, subgroup analyses focused on gender to identify structurally vulnerable groups for policy targeting. Education level was considered relevant but was not selected as the primary stratification variable in the present analysis, although it could be important to consider education level in designing an intervention, particularly those aimed at improving food literacy. Consequently, elderly men living alone, who can be more feasibly identified and reached through behavior-based interventions, should be prioritized in policies aimed at addressing nutritional vulnerability in Seoul.

Food literacy was identified as a key dietary characteristic associated with nutritional vulnerability. Although the cross-sectional design of this study precludes causal inference, there is a need for policies that support the elderly with low food literacy in practicing healthy diet. In particular, food environment policies should ensure that the elderly have convenient access to balanced meals. Such conditions are especially important for elderly men living alone, who have been identified as a priority group for intervention. Given traditional gender norms and limited cooking experience (35), they are likely to face greater challenges in meal preparation, highlighting the need for tailored support.

In addition to these policy recommendations, future research should explore how independent variables are associated with each domain of the NQ-E (balance, moderation, and practice) to clarify which factors are primarily related to food intake and which to dietary behaviors. Such research would provide valuable evidence to design more tailored and effective interventions and to reduce health inequalities among elderly populations.

This study has several limitations that should be considered. To begin with, the cross-sectional design limits the ability to establish causal relationships between sociodemographic or dietary characteristics and nutritional vulnerability. Moreover, due to the nature of the 2024 Seoul Food Survey, this study did not include detailed quantitative data on actual nutrient intake or specific food group consumption among the elderly, restricting the ability to comprehensively assess nutritional deficiencies or dietary patterns within the nutritionally vulnerable group. In addition, because the study relied on self-reported survey data, recall bias may have attenuated the observed associations toward the null. Moreover, although some potential confounders, such as smoking, physical activity, and chronic disease status, were not available in the dataset, residual confounding is unlikely to fully account for the observed associations.

Despite these limitations, this study demonstrates several notable contributions. By applying the NQ-E to the most recent Seoul Food Survey data, we replicate the identification of nutritionally vulnerable elderly individuals and thereby reinforce the continued relevance and applicability of this Korea-specific assessment tool in a rapidly aging urban context. Furthermore, the integration of food literacy, digital food literacy, and food environment satisfaction into the analysis extends previous research by providing additional evidence on characteristics associated with nutritional vulnerability among the elderly. Taken together, these contributions offer timely, policy-relevant insights that may inform the development of targeted nutrition-support strategies for the elderly in Seoul.

In summary, approximately 19% of the elderly population in Seoul were classified as nutritionally vulnerable. Among sociodemographic characteristics, living alone was significantly associated with increased odds of nutritional vulnerability among elderly men, highlighting the need to prioritize this group in future interventions. In terms of dietary characteristics, food literacy emerged as a key characteristic associated with nutritional vulnerability across the total elderly population. These findings suggest that food literacy should be explored as a potential strategy to address nutritional vulnerability among the elderly, with particular emphasis on elderly men living alone, who face greater challenges in meal preparation due to traditional gender norms and limited cooking experience. Building on this evidence, the findings of this study provide essential baseline data to guide policies aimed at improving the dietary practices of the nutritionally vulnerable elderly in Seoul, ultimately contributing to better health outcomes and quality of life among the city’s elderly population.

## Data Availability

Publicly available datasets were analyzed in this study. This data can be found here: the data analyzed in this study are publicly available from the Seoul Open Data Plaza at the following URL: https://data.seoul.go.kr/dataList/OA-22263/F/1/datasetView.do.
