# Supplementary material for: Nutritional vulnerability and its associated characteristics among the elderly in Seoul: analysis of data from the Seoul food survey 2024
Source: Front Nutr. 2026 Feb 26;13:1662335. doi: 10.3389/fnut.2026.1662335 (PMC12979154; doi:10.3389/fnut.2026.1662335)
Supplement: Supplementary file 1 [file Data_Sheet_1.pdf]

Supplement 1. 2024 Seoul Food Survey questionnaire (excerpt of items used for analysis)

A. Food Intake

The following questions concern your **food intake**. Please answer while considering your food consumption **during the past month**.

1 A How often do you usually eat **breakfast in a week**?

① Hardly ever ② 1–2 times per week ③ 3–4 times per week

④ 5–6 times per week ⑤ Every day

2 A Please indicate how often you have consumed the following foods **during the past month**. Include not only food consumed at home but also meals from institutional dining and eating out.

Item	Once every two weeks or less	1–3 times per week	4–6 times per week	Once daily	Twice daily or more
1) Fruit	①	②	③	④	⑤
2) Raw vegetables (including lettuce wraps, cucumbers, carrots, salads)	①	②	③	④	⑤
3) Vegetable side dishes (including seaweed, mushrooms, seasoned vegetables, roasted seaweed; excluding kimchi and pickled vegetables)	①	②	③	④	⑤
4) Kimchi and pickled vegetables	①	②	③	④	⑤
5) Milk or dairy products (e.g., cheese, yogurt)	①	②	③	④	⑤
6) Beans or tofu (including soy milk)	①	②	③	④	⑤
7) Spicy or salty soup-based foods (e.g., ramen, stews, soups, tteokbokki with broth)	①	②	③	④	⑤
**Item**	**Hardly ever**	**1–3 times per week**	**4–6 times per week**	**Once daily**	**Twice daily or more**
8) Whole grains or mixed grains (e.g., brown rice, multigrain rice, whole wheat bread)	①	②	③	④	⑤

Item		Hardly ever	Once every two weeks	1–3 times per week	4–6 times per week	Once daily or more
9) Red meat (e.g., beef, pork)		①	②	③	④	⑤
10) Processed meat (e.g., ham, sausage, bacon)		①	②	③	④	⑤
11) Eggs (including quail eggs)		①	②	③	④	⑤
[For respondents under age 65]	12) Fish	①	②	③	④	⑤
[For respondents aged 65 and older]	12) Fish or shellfish (including squid, oysters, shrimp, etc.)	①	②	③	④	⑤
13) Nuts (e.g., walnuts, pine nuts, almonds)		①	②	③	④	⑤
14) Fatty breads (e.g., fried patties cream bread) or snack foods (e.g., potato chips, sweet potato chips)		①	②	③	④	⑤
15) Fast foods (e.g., pizza, hamburgers, fried chicken)		①	②	③	④	⑤

Item	Hardly ever	1–3 times per week	4–6 times per week	1–2 times per day	3 or more times per day
16) Snacks high in sugar (e.g., instant coffee mix, sweetened beverages candies)	①	②	③	④	⑤

Item	Hardly ever	1–2 cups per day	3–4 cups per day	5–6 cups per day	7–8 cups per day or more
17) Water	①	②	③	④	⑤

3 A **How many kinds of vegetables (excluding kimchi)** did you usually eat in one meal **during the past month?**

① Hardly ever ② 1 kind ③ 2 kinds ④ 3 kinds ⑤ 4 kinds or more

4 A For men, how often do you drink **7 or more glasses of alcohol (equivalent to about 5 cans of beer)**, and for women, **5 or more glasses of alcohol (equivalent to about 3 cans of beer)**?

① Not at all in the past year ② Less than once a month ③ About once a month

④ 2–4 times per month ⑤ 2–3 times per week ⑥ 4 or more times per week

5 A **During the past month,** how often did you **binge eat or overeat?**

① Hardly ever ② Once a month ③ Once every two weeks

④ Once a week ⑤ 3–4 times per week ⑥ Once or more per day

B. Dietary practice

The following questions concern your **dietary life**. Depending on the question, please answer while considering either your **past year** or your **current** dietary practices.

6 B Do you usually make an effort to maintain **a healthy diet**?

① Not at all ② Hardly ever ③ Neutral
④ To some extent ⑤ Very much

7 B How would you describe your overall current health status?

① Very poor ② Poor ③ Neutral ④ Good ⑤ Very good

8 B How often do you usually **feel depressed**?

① Always ② Often ③ Neutral
④ Rarely ⑤ Never

9 B How well do you usually sleep?

① Very poorly ② Poorly ③ Neutral
④ Well ⑤ Very well

10 B How satisfied are you with your current dietary life?

① Very dissatisfied ② Dissatisfied ③ Neutral ④ Satisfied ⑤ Very satisfied

11 B How important is dietary life to your happiness?

① Not important at all ② Not important ③ Neutral ④ Important ⑤ Very important

12 B Do you **wash your hands before eating food**?

① Never ② Rarely ③ Neutral
④ Usually ⑤ Always

13 B **[For respondents under age 65]**
When eating out or purchasing processed foods, do you check the nutrition label?

① Never ② Rarely ③ Neutral
④ Usually ⑤ Always

14 B **[For respondents aged 65 and older]**
When purchasing (processed) foods, do you check the **expiration date** or the **nutrition label**?

① Never ② Rarely ③ Neutral

④ Often ⑤ Always

15 B **[For respondents aged 65 and older]** How uncomfortable is it for you **to chew food** due to problems with your teeth, dentures, or gums?

① Very uncomfortable ② Somewhat uncomfortable ③ Neutral

④ Not very uncomfortable ⑤ Not uncomfortable at all

16 B For each of the following statements, please select the option that best describes you.

Item	Strongly disagree	Disagree	Neutral	Agree	Strongly agree
1) I know about the various food groups needed to compose a nutritionally balanced meal.	①	②	③	④	⑤
2) I know how to divide and store ingredients that are not used immediately.	①	②	③	④	⑤
3) I know the appropriate storage methods for different foods (e.g., freezing, refrigeration, room temperature).	①	②	③	④	⑤

17 B For each of the following statements, please select the option that best describes you.

Item	Strongly disagree	Disagree	Neutral	Agree	Strongly agree
1) I can understand food information on processed products (e.g., list of ingredients, nutrition label).	①	②	③	④	⑤
2) I can follow simple cooking instructions.	①	②	③	④	⑤
3) I can prepare a meal without much difficulty.	①	②	③	④	⑤
4) I can understand food safety information presented in media (e.g., food poisoning, radioactive contamination, hygiene of imported foods).	①	②	③	④	⑤
5) I can evaluate whether food advertisements claiming health benefits are accurate.	①	②	③	④	⑤

18 B For each of the following statements, please select the option that best describes you.

Item	Strongly disagree	Disagree	Neutral	Agree	Strongly agree
1) I make a shopping list before buying groceries.	①	②	③	④	⑤
2) I check food information (e.g., list of ingredients, nutrition label) when purchasing processed foods.	①	②	③	④	⑤
3) I check the country of origin when purchasing foods.	①	②	③	④	⑤
4) I wash my hands thoroughly before cooking.	①	②	③	④	⑤
5) I check the sanitary conditions when visiting restaurants or food service establishments.	①	②	③	④	⑤
6) I try to consume a variety of food groups (grains, fish/meat/eggs, vegetables, fruits, dairy products) in a balanced way.	①	②	③	④	⑤

19 B For each of the following statements, please select the option that best describes you.

Item	Strongly disagree	Disagree	Neutral	Agree	Strongly agree
1) Cooking is an enjoyable activity.	①	②	③	④	⑤
2) When eating, I focus on the food without doing other activities.	①	②	③	④	⑤
3) When eating, I savor various sensory aspects such as visual appearance, aroma, taste, and texture.	①	②	③	④	⑤
4) When facing food, I think about the process it went through before reaching the table and feel grateful.	①	②	③	④	⑤
5) I enjoy sharing meals with or eating together with family, acquaintances, or neighbors.	①	②	③	④	⑤
6) I enjoy talking about food with people around me.	①	②	③	④	⑤
7) I am interested in foods from diverse cultures.	①	②	③	④	⑤
8) I believe that enjoying traditional foods is important for our dietary life and culture.	①	②	③	④	⑤

20 B For each of the following statements, please select the option that best describes you.

Item	Strongly disagree	Disagree	Neutral	Agree	Strongly agree
1) I know why choosing seasonal foods is good for the environment.	①	②	③	④	⑤
2) I know why animal welfare* should be considered when purchasing meat and eggs.	①	②	③	④	⑤
3) I know why fair trade products* should be chosen.	①	②	③	④	⑤

2) Animal welfare-certified products: Products produced with consideration for animals as food, minimizing pain, stress, and suffering caused by humans.
3) Fair trade products: Products produced by paying fair compensation to producers in developing countries, supporting their economic independence and sustainable development

21 B For each of the following statements, please select the option that best describes you.

Item	Strongly disagree	Disagree	Neutral	Agree	Strongly agree
1) I am interested in food purchased directly from producers.	①	②	③	④	⑤
2) Choosing eco-friendly agricultural products (e.g., organic, pesticide-free) is important for ecological and environmental conservation.	①	②	③	④	⑤
3) I am interested in urban farming (e.g., home gardening, weekend farms).	①	②	③	④	⑤
4) Reducing meat consumption or practicing vegetarianism helps slow down climate change.	①	②	③	④	⑤
5) Regardless of economic status, everyone should have sufficient access to quality food.	①	②	③	④	⑤
6) Rural areas and farmers are important for the sustainability* of our society's food system.	①	②	③	④	⑤

6) Food system sustainability: Ensuring the stable and continuous provision of food through the balance of environmental, economic, and social factors.

22 B For each of the following statements, please select the option that best describes you.

Item	Strongly disagree	Disagree	Neutral	Agree	Strongly agree
1) I make an effort to reduce food waste in my daily life.	①	②	③	④	⑤
2) I make an effort to reduce packaging waste (e.g., take-out drinks, delivered food) in my daily life.	①	②	③	④	⑤

23 B For each of the following statements, please select the option that best describes you.

Item	Strongly disagree	Disagree	Neutral	Agree	Strongly agree
1) I can purchase food from an online shopping mall*.	①	②	③	④	⑤
2) I can order food using an unmanned ordering machine (kiosk) at restaurants.	①	②	③	④	⑤
3) I can order food using a food delivery application (app).	①	②	③	④	⑤

1) Online shopping mall: A shopping platform accessible via computer or smartphone (excluding phone orders).

C. Food Purchasing

The following questions concern your **food purchasing.** Please answer while considering your current food purchasing situation.

24 C For each of the following statements, please select the option that best describes you.

Item	Strongly disagree	Disagree	Neutral	Agree	Strongly agree	No purchase experience
1) There are sufficient grocery stores in my neighborhood offering a variety of healthy foods (e.g., fruits, vegetables).	①	②	③	④	⑤	⑨
2) Grocery stores that offer a variety of healthy foods are located close to my home.	①	②	③	④	⑤	
3) Foods sold at grocery stores in my neighborhood are reasonably priced for me to purchase.	①	②	③	④	⑤	
4) The services of grocery stores in my neighborhood (e.g., small-quantity purchase, delivery availability, business hours) are convenient to use.	①	②	③	④	⑤	
5) Foods sold at grocery stores in my neighborhood are fresh and of good quality.	①	②	③	④	⑤	

DQ. Respondent Characteristics

25 D Do you **mainly** purchase food for your household?

① Yes ② No

26 D Do you **mainly** prepare meals for your household?

① Yes ② No

27 D What is your occupation?
If you have more than one job, please respond based on your primary occupation.

① Manager ② Professional and related worker
③ Clerical worker ④ Service worker
⑤ Sales worker ⑥ Agricultural, forestry, or fishery worker
⑦ Skilled worker and related trades worker
⑧ Equipment, machine operation, or assembly worker
⑨ Elementary (unskilled) worker

→ Item To DQ4

⑩ Student ⑪ Homemaker
⑫ Unemployed

→ Item To DQ5

⑬ Other ( )

→ Item To DQ4

28 D **[For respondents who are employed only]**
Which of the following best describes your employment status?

① Regular wage worker ② Temporary or daily wage worker

③ Self-employed without employees ④ Self-employed with employees

⑤ Unpaid family worker ⑥ Other (______________________)

29 D What is your highest level of education completed?

① Less than elementary school ② Elementary school graduate

③ Middle school graduate ④ High school graduate

⑤ Currently enrolled in college/university ⑥ College/university graduate or higher

30 D Based on your household's economic and social conditions, where do you think your household belongs in our society?

0	1	2	3	4	5	6	7	8	9	10
Low class					Mid class					High class

31 D **[For household heads only]**
What is your household's average monthly income (before tax deductions)?

① Less than 500,000 KRW ② 500,000 – Less than 1,000,000 KRW

③ 1,000,000 – Less than 1,500,000 KRW ④ 1,500,000 – Less than 2,000,000 KRW

⑤ 2,000,000 – Less than 2,500,000 KRW ⑥ 2,500,000 – Less than 3,000,000 KRW

⑦ 3,000,000 – Less than 3,500,000 KRW ⑧ 3,500,000 – Less than 4,000,000 KRW

⑨ 4,000,000 – Less than 4,500,000 KRW ⑩ 4,500,000 – Less than 5,000,000 KRW

⑪ 5,000,000 – Less than 5,500,000 KRW ⑫ 5,500,000 – Less than 6,000,000 KRW

⑬ 6,000,000 – Less than 6,500,000 KRW ⑭ 6,500,000 – Less than 7,000,000 KRW

⑮ 7,000,000 – Less than 7,500,000 KRW ⑯ 7,500,000 – Less than 8,000,000 KRW

⑰ 8,000,000 – Less than 8,500,000 KRW ⑱ 8,500,000 – Less than 9,000,000 KRW

⑲ 9,000,000 – Less than 9,500,000 KRW ⑳ 9,500,000 – Less than 10,000,000 KRW

㉑ 10,000,000 KRW or more (_________ KRW)

We sincerely appreciate your valuable time and cooperation in completing this survey.

Supplement 2. Nutrition Quotient for the Elderly (NQ-E) measurement items in the 2024 Seoul Food Survey

Domain (No. of items)	No.	Question	Response Options
Balance (8)	A2-1	How frequently do you consume fruit?	① Once every two weeks or less<br>② 1–3 times per week<br>③ 4–6 times per week<br>④ Once daily<br>⑤ Twice daily or more
	A2-5	How frequently do you consume milk or dairy products (e.g., cheese, yogurt)?	① Once every two weeks or less<br>② 1–3 times per week<br>③ 4–6 times per week<br>④ Once daily<br>⑤ Twice daily or more
	A2-6	How frequently do you consume beans or tofu (including soy milk)?	① Once every two weeks or less<br>② 1–3 times per week<br>③ 4–6 times per week<br>④ Once daily<br>⑤ Twice daily or more
	A2-8	How frequently do you consume whole grains or mixed grains (e.g., brown rice, multigrain rice, whole wheat bread)?	① Hardly ever<br>② 1–3 times per week<br>③ 4–6 times per week<br>④ Once daily<br>⑤ Twice daily or more
	A2-11	How frequently do you consume eggs (including quail eggs)?	① Hardly ever<br>② Once every two weeks<br>③ 1–3 times per week<br>④ 4–6 times per week<br>⑤ Once daily or more
	A2-12	How frequently do you consume fish or shellfish (including squid, oysters, shrimp, etc.)?	① Hardly ever<br>② Once every two weeks<br>③ 1–3 times per week<br>④ 4–6 times per week<br>⑤ Once daily or more
	A2-13	How frequently do you consume nuts (e.g., walnuts, pine nuts, almonds)?	① Hardly ever<br>② Once every two weeks<br>③ 1–3 times per week<br>④ 4–6 times per week<br>⑤ Once daily or more
	A2-17	On average, how many cups of water do you consume per day?	① Hardly ever<br>② 1–2 cups daily<br>③ 3–4 cups daily<br>④ 5–6 cups daily<br>⑤ 7–8 cups daily or more
Moderation (2)	A2-16	How frequently do you consume snacks high in sugar (e.g., instant coffee mix, sweetened beverages, candies)?	① Hardly ever<br>② 1–3 times per week<br>③ 4–6 times per week<br>④ 1–2 times daily<br>⑤ 3 times daily or more
	A2-14	How frequently do you consume fatty breads (e.g., fried patties, cream bread) or snack foods (e.g., potato chips, sweet potato chips)?	① Hardly ever<br>② Once every two weeks<br>③ 1–3 times per week<br>④ 4–6 times per week<br>⑤ Once daily or more
Practice (7)	B4	Do you usually make an effort to maint ain a healthy diet?	① Not at all<br>② Harldy ever<br>③ Neutral<br>④ To some extent<br>⑤ Very much
	B15	When purchasing processed foods, do y ou check the expiration date or nutritio n label?	① Never<br>② Rarely<br>③ Neutral<br>④ Often

			⑤ Always
	B13	Do you wash your hands before eating food?	① Never<br>② Rarely<br>③ Neutral<br>④ Often<br>⑤ Always
	B16	How uncomfortable is it for you to che w food due to problems with your teet h, dentures, or gums?	① Very uncomfortable<br>② Somewhat uncomfortable<br>③ Neutral<br>④ Not very uncomfortable<br>⑤ Not uncomfortable at all
	B6	How often do you usually feel depresse d?	① Always<br>② Often<br>③ Neutral<br>④ Rarely<br>⑤ Never
	B7	How well do you usually sleep?	① Very poorly<br>② Poorly<br>③ Neutral<br>④ Well<br>⑤ Very well
	B5	How would you describe your overall c urrent health status?	① Very poor<br>② Poor<br>③ Neutral<br>④ Good<br>⑤ Very good

No. = questionnaire item number

Supplement 3. Food literacy measurement items in the 2024 Seoul Food Survey

Section (No. of items)	No.	Question
Health (14)	B17-1	I know about the various food groups needed to compose a nutrit ionally balanced meal.
	B17-2	I know how to divide and store ingredients that are not used im mediately.
	B17-3	I know the appropriate storage methods for different foods (e.g., f reezing, refrigeration, room temperature).
	B18-1	I can understand food information on processed products (e.g., list of ingredients, nutrition label).
	B18-2	I can follow simple cooking instructions.
	B18-3	I can prepare a meal without much difficulty.
	B18-4	I can understand food safety information presented in media (e.g., food poisoning, radioactive contamination, hygiene of imported foo ds).
	B18-5	I can evaluate whether food advertisements claiming health benefit

Section (No. of items)	No.	Question
		s are accurate.
	B19-1	I make a shopping list before buying groceries.
	B19-2	I check food information (e.g., list of ingredients, nutrition label) when purchasing processed foods.
	B19-3	I check the country of origin when purchasing food.
	B19-4	I wash my hands thoroughly before cooking.
	B19-5	I check the sanitary conditions when visiting restaurants or food s ervice establishments.
	B19-6	I try to consume a variety of food groups (grains, fish/meat/eggs, vegetables, fruits, dairy products) in a balanced way.
Enjoyment (8)	B20-1	Cooking is an enjoyable activity.
	B20-2	When eating, I focus on the food without doing other activities.
	B20-3	When eating, I savor various sensory aspects such as visual appea rance, aroma, taste, and texture.
	B20-4	When facing food, I think about the process it went through befo re reaching the table and feel grateful.
	B20-5	I enjoy sharing meals with or eating together with family, acquain tances, or neighbors.
	B20-6	I enjoy talking about food with people around me.
	B20-7	I am interested in foods from diverse cultures.
	B20-8	I believe that enjoying traditional foods is important for our dietar y life and culture.
Value (11)	B21-1	I know why choosing seasonal foods is good for the environment.
	B21-2	I know why animal welfare should be considered when purchasin g meat and eggs.
	B21-3	I know why fair-trade products should be chosen.
	B22-1	I am interested in food purchased directly from producers.
	B22-2	Choosing eco-friendly agricultural products (e.g., organic, pesticide-free) is important for ecological and environmental conservation.
	B22-3	I am interested in urban farming (e.g., home gardening, weekend farms).
	B22-4	Reducing meat consumption or practicing vegetarianism helps slow down climate change.
	B22-5	Regardless of economic status, everyone should have sufficient acc ess to quality food.
	B22-6	Rural areas and farmers are important for the sustainability of our society’s food system.
	B23-1	I make an effort to reduce food waste in my daily life.
	B23-2	I make an effort to reduce packaging waste (e.g., take-out drinks, delivered food) in my daily life.

**Section** (No. of items)	**No.**	**Question**

No. = questionnaire item number

All items were rated on a 5-point Likert scale (1 = strongly disagree, 2 = disagree, 3 = neutral, 4 = agr ee, 5 = strongly agree) in response to the instruction: ‘Please select the option that best describes you..

Supplement 4. Digital food literacy measurement items in the 2024 Seoul Food Survey

**Section** (No. of items)	**No.**	**Question**
Digital food literacy	B24-1	I can purchase food from an online shopping mall.
	B24-2	I can order food using an unmanned ordering machine (kiosk) at re staurants.
	B24-3	I can order food using a food delivery application (app).

No. = questionnaire item number

All items were rated on a 5-point Likert scale (1 = strongly disagree, 2 = disagree, 3 = neutral, 4 = agre e, 5 = strongly agree) in response to the instruction: ‘Please select the option that best describes you..

Supplement 5. Digital food literacy measurement items in the 2024 Seoul Food Survey

**Section** (No. of items)	**No.**	**Question**
Food environment satisfaction	C5-1	(Availability) There are sufficient grocery stores in my neighborhoo d offering a variety of healthy foods (e.g., fruits, vegetables).
	C5-2	(Accessibility) Grocery stores that offer a variety of healthy foods are located close to my home.
	C5-3	(Affordability) Foods sold at grocery stores in my neighborhood are reasonably priced for me to purchase.
	C5-4	(Convenience) The services of grocery stores in my neighborhood (e.g., small-quantity purchase, delivery availability, business hours) a re convenient to use.
	C5-5	(Acceptability) Foods sold at grocery stores in my neighborhood ar e fresh and of good quality.

No. = questionnaire item number

All items were rated on a 5-point Likert scale (1 = strongly disagree, 2 = disagree, 3 = neutral, 4 = agre e, 5 = strongly agree) in response to the instruction: ‘Please select the option that best describes you..
