# Supplementary material for: Nutritional vulnerability and its associated characteristics among the elderly in Seoul: analysis of data from the Seoul food survey 2024
Source: Front Nutr. 2026 Feb 26;13:1662335. doi: 10.3389/fnut.2026.1662335 (PMC12979154; doi:10.3389/fnut.2026.1662335)
Supplement: Supplementary file 2 [file Data_Sheet_2.pdf]

Appendix A

Table 1. Domain-specific NQ-E Scores among the elderly in Seoul by NQ-E Grade

Items	Total (n=720)	NQ-E grade			*p*-value[1)]
		Low (n=83)	Medium (n=321)	High (n=316)	
NQ-E	**51.51±0.3**	**37.51^{c}±0.6**	**51.77^{b}±0.4**	**65.23^{a}±0.4**	**<0.001**
**Balance[2)]**	**45.96±0.6**	**28.96^{c}±1.4**	**46.40^{b}±0.9**	**62.51^{a}±1.0**	**<0.001**
Intake frequency of fruits	43.65±1.2	20.19^{c}±2.3	44.53^{b}±2.1	66.24^{a}±1.7	<0.001
Intake frequency of milk or dairy products	35.92±1.3	15.53^{c}±2.6	36.70^{b}±1.8	55.54^{a}±2.0	<0.001
Intake frequency of fish or shellfish	39.15±0.9	24.68^{c}±2.0	41.29^{b}±1.7	51.48^{a}±1.2	<0.001
Intake frequency of eggs	54.60±2.2	43.19^{b}±5.5	52.85^{b}±2.1	67.77^{a}±1.5	<0.001
Intake frequency of beans or bean products	41.57±1.1	25.01^{c}±2.0	44.48^{b}±1.9	55.22^{a}±2.0	<0.001
Intake frequency of nuts	34.03±1.5	12.79^{c}±2.4	33.85^{b}±2.2	55.44^{a}±2.8	<0.001
Intake frequency of whole grains or mixed grains	60.39±2.0	41.15^{c}±4.8	65.54^{b}±2.4	74.46^{a}±2.8	<0.001
Intake frequency of water	70.72±1.7	68.88^{b}±3.8	64.66^{b}±2.0	78.61^{a} ±1.5	<0.001
**Moderation[3)]**	**52.41±1.6**	**51.10^{ab}±3.9**	**48.95^{b}±1.8**	**57.17^{a}±2.4**	**0.022**
Intake frequency of sweetened snacks or beverages	49.58±1.7	47.75^{ab}±4.3	45.99^{b}±1.9	55.00^{a}±2.7	0.022
Intake frequency of fatty baked products or snacks	73.94±1.4	76.57±2.7	71.56±2.2	73.70±2.1	0.333
**Practice[4)]**	**60.00±0.8**	**47.11^{c}±1.3**	**61.05^{b}±0.9**	**71.84^{a}±1.4**	**<0.001**
Efforts to maintain a healthy diet	61.95±1.3	45.12^{c}±3.4	64.74^{b}±1.3	76.00^{a}±1.6	<0.001
Expiration date and nutrition labeling check	50.33±1.8	32.18^{b}±3.8	56.22^{a}±2.3	62.60^{a}±1.9	<0.001
Washing hands practices before eating meals	75.46±1.1	68.61^{c}±2.5	75.15^{b}±1.3	82.61^{a}±1.4	<0.001
Difficulties in chewing foods	55.28±1.5	41.82^{c}±3.0	53.56^{b}±2.3	70.45^{a}±1.9	<0.001
Depressed condition	60.31±1.4	50.53^{c}±2.6	60.58^{b}±2.2	69.83^{a}±2.1	<0.001
Degree of sound sleep	60.49±1.4	48.81^{b}±3.1	63.59^{a}±1.6	69.07^{a}±2.2	<0.001
Level of awareness of one's own health	55.09±1.4	40.57^{c}±2.8	54.43^{b}±1.5	70.27^{a}±2.2	<0.001

NQ-E, Nutrition Quotient for the Elderly

Values are presented as mean ± standard error.

All weighted models accounted for the complex sampling design of the Seoul Food Survey 2024.

[1)]Analysis of variance. Complex sample analysis with Bonferroni post-hoc test (a > b > c).

[2)]Balance domain of the NQ-E assesses dietary diversity based on the intake frequency of fruits, dairy products, fish or shellfish, eggs, beans, nuts, mixed grains, and water. Higher intake frequency results in higher scores (0–100 scale).

[3)]Moderation domain of the NQ-E evaluates the intake frequency of sugar-sweetened snacks/beverages and fatty baked products/snacks. Lower intake frequency results in higher scores (0–100 scale).

[4)]Practice domain of the NQ-E assesses desirable eating behaviors, including efforts to maintain a healthy diet, checking expiration dates and nutrition labels, handwashing before meals, chewing comfort, absence of depression, sleep quality, and self-perceived health. Higher scores reflect more favorable practices (0–100 scale).
